# Stress state measured at ~7 km depth in the Tarim Basin, NW China

**DOI:** 10.1038/s41598-017-04516-9

**Published:** 2017-07-03

**Authors:** Dongsheng Sun, Hiroki Sone, Weiren Lin, Junwen Cui, Bizhu He, Haitao Lv, Zicheng Cao

**Affiliations:** 1 Institute of Geomechanics, Chinese Academy of Geological Sciences, Beijing, 100081 China; 2 Department of Civil and Environmental Engineering, Geological Engineering Program, University of Wisconsin-Madison, Madison, WI 53706 USA; 3 Graduate School of Engineering, Kyoto University, Kyoto, 615-8540 Japan; 4 Kochi Institute for Core Sample Research, Japan Agency for Marine-Earth Science and Technology, Nankoku, 783-8502 Japan; 5 Institute of Geology, Chinese Academy of Geological Sciences, Beijing, 100037 China; 6 Tarim Oil-field Company, PetroChina, Korla, Xinjiang 841000 China

## Abstract

The *in-situ* stress state in the Tarim Basin, Northwest China, down to 7 km depth is constrained using the anelastic strain recovery (ASR) method and wellbore failure analysis. Results are consistent between the two methods, and indicate that the maximum principal stresses (σ_1_) are close to vertical and the intermediate and minimum principal stresses (σ_2_ and σ_3_) are approximately horizontal. The states of stress at the studied wellbore is in the normal faulting stress regime within the Tarim Basin rather than in the compressional tectonic stress regime as in the periphery of the Tarim Basin, which explains the presence of the normal faults interpreted in 3-D seismic profiles collected from adjacent areas. Our results demonstrate that the ASR method can be used for rocks recovered from depths as deep as 7 km to recover reliable stress state information. The *in-situ* stress measurement results revealed in this paper will help future development of the petroleum resources and kinematics study in the Tarim Basin.

## Introduction

**Figure 1:**
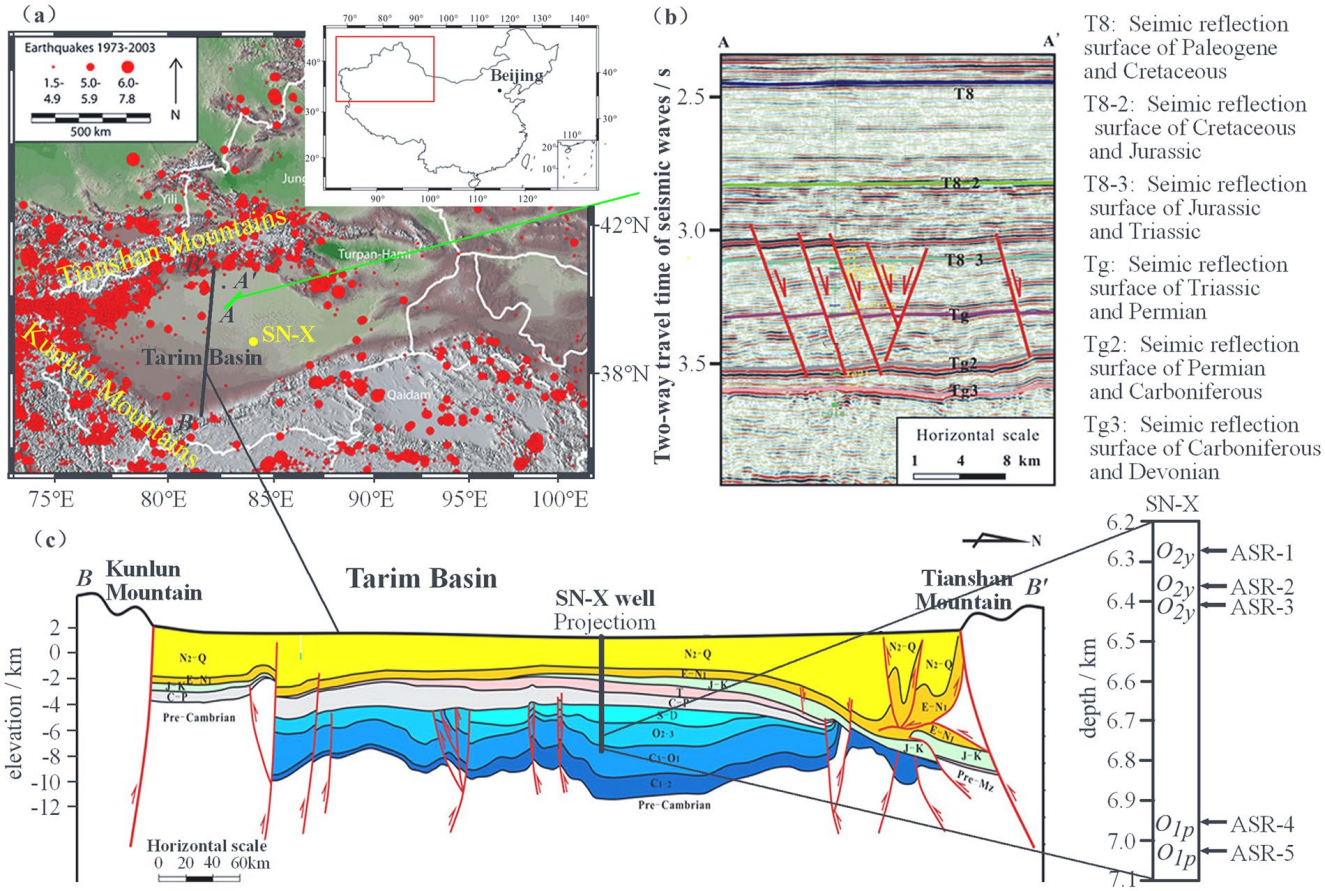
(**a**) Earthquake epicenters (red circles, scaled according to magnitude) from 1973 to 2003 around the Tarim Basin^3^, where the yellow circle is the SN-X well position, (**b**) Seismic profile along the transect A-A’ in panel (a) in the Lunnan Lower Uplift located in a Meso-Cenozoic normal fault zone, where the red lines represent the normal faults interpreted from 3D seismic data^5^, (**c**) A tectonic profile across the Tarim Basin from south to north along the transect B-B’ in panel (a)^5^, on which the location of the SN-X well is projected. Coring interval of SN-X is about 6200–7100 m in the Lower Ordovician Yingshan formation (O_*2y*_) and the Lower Ordovician Penglaiba formation (O_*1p*_). Depths of the ASR samples are also shown to the right.

The Tarim Basin is one of the largest Meso-Cenozoic hydrocarbon-bearing Basins in Central Asia located in Northwest China between the Tianshan and Kunlun Mountains. The Tarim block has experienced a complex tectonic history during its formation and evolution process^1^. Himalayan collisional orogeny was the massive neo-tectonic movement which controlled the strong regional compressional tectonic regime of the Tarim block during the Late Cenozoic era^2^. Thus, the present-day *in-situ* stress state of Tarim Basin is also expected to be dominated by the Himalayan orogeny, promoting thrust faulting. Accordingly, the peripheries of the Tarim Basin are seismically active zones exhibiting pronounced intraplate deformation (Fig. 1a). However, the interiors of Tarim Basin records limited seismic activity and show relatively minor deformation^3^. While the compressional thrust structures at the periphery generally become the focus of investigation, a large number of small normal faults associated with the Paleozoic-Cenozoic extensional structures are also interpreted from seismic data which have triggered discussion on why such structures exist and what marks the transition in the stress state^4, 5^ (Fig. 1b).

We studied wellbore data and core samples recovered from the SN-X ultra-deep vertical well (deviation <2 degrees) located at the Shunnan area (Fig. 1a), one of the key focus areas in the Tarim Basin^6–8^ for potential oil and gas production. The Ordovician Yingshan Formation, buried at 6000–7000 m depth, is the key reservoir of the Shunnan oil-field, which consists of intraclastic shoal limestone with tiny vugs and karst holes. The cores were recovered from the SN-X well from about 6200–7100 m depth within the Lower Ordovician Yingshan formation (*O*_*2y*_) and the Lower Ordovician Penglaiba formation (*O*_*1p*_)^7^ (Fig. 1c). The formation temperature at the coring interval was 170–190 °C. Five cores were retrieved from different depths in the SN-X well for ASR measurements. These samples mostly consist of calcite with a minor amount of dolomite. Descriptions of each sample are given in Table 1.

**Table 1:** Basic properties and depths of the core samples.

Core Num.	Formation	Classification	Top depth (m)	Bottom depth (m)	Natural density (g/cm^3^)	Porosity (%)	Mineral composition (%)	Period^c^ (hour)	Azimuth^d^ (°)
ASR-1	Yingshan formation	Micritic limestone	6293.09	6293.29	2.652	1.25^a^	Calcite: 100	65	82
ASR-2			6369.54	6369.71	2.646			40	125
ASR-3			6410.38	6410.56	2.649			80	91
ASR-4	Penglaiba formation	Dolomitic limestone	6955.41	6955.64	2.657	1.15^b^	Calcite: 60 Dolomite: 40	48	155
ASR-5			7022.28	7022.42	2.669		Calcite: 70 Dolomite: 30	96	2

^a^Average value of 852samples, ^b^Average value of 460 samples^6^, ^c^The time span between stress release by coring bit penetration and start of ASR measurement; ^d^Azimuth denotes the angle between true North and the reference line direction of core.

Measuring and constraining *in-situ* stress becomes more difficult as depth increases. Generally, hydraulic fracturing (HF) is the best known and most direct method for *in-situ* stress magnitude estimation in the subsurface^9, 10^. However, the applicability of the HF method may be limited at great depths due to the temperature and pressure limits of the packer material. Observation of wellbore failures by borehole image is an indirect strategy to infer subsurface stress magnitudes which can complement HF stress measurements. But wellbore failures are not always prevalent in image logs and only impose limits on the range of possible stress magnitudes. Up to date, the maximum depth at which hydraulic fracturing was successfully employed for *in-situ* stress measurements is 9 km, and the maximum depth of stress profiles constrained by borehole breakouts and drilling-induced tensile fractures (DITFs) is 8 km^11^.

Anelastic strain recovery (ASR)^12, 13^, on the other hand, is a core-based method which relies on the measurement of the time-dependent strain recovery of rocks due to the sudden release of *in-situ* stress. There are strict requirements about sample isotropy and assumptions on certain rock behavior, but there is a relatively explicit theoretical basis and measurements can be conducted relatively easily as long as oriented core samples are available. Merits of the ASR method are that it is technically simple and can recover the full three-dimensional *in-situ* stress tensor^14–16^. The restriction for the application of ASR method is obviously that it requires measurable amounts of recovery strain. This translates to depth limits of application since it takes longer for deeper core samples to reach the surface and strain recovery rates of rocks decrease with time. Therefore measurements of anelastic recovery strain should be conducted as soon as possible after the cores have been released from the *in-situ* stress. On the other hand, the magnitude of recovery strains of a core may increase with depth because of the larger magnitude of stress release (i.e. stress magnitudes are generally proportional to depth). Therefore, depth can also work to our advantage at times for ASR measurements and the depth limit of application of the ASR method is not necessarily determined solely by the time required for core recovery.

In this study, we first present the orientations and magnitudes of *in-situ* stresses at ~7 km depth estimated by the ASR method. Then information about the DITFs observed in borehole image logs, as well as some drilling engineering parameters, are used to constrain the possible range of *in-situ* stress magnitudes. The results of the ASR method and the wellbore failure information are generally consistent and allow us to evaluate the reliability of the methods. Our results reveal that the current stress state in the interior of the Tarim Basin is a normal faulting stress regime, in contrast to the compressional tectonic stress regime developed in the periphery of the Tarim Basin^2^.

## Results

### The anelastic recovery strain

**Figure 2:**
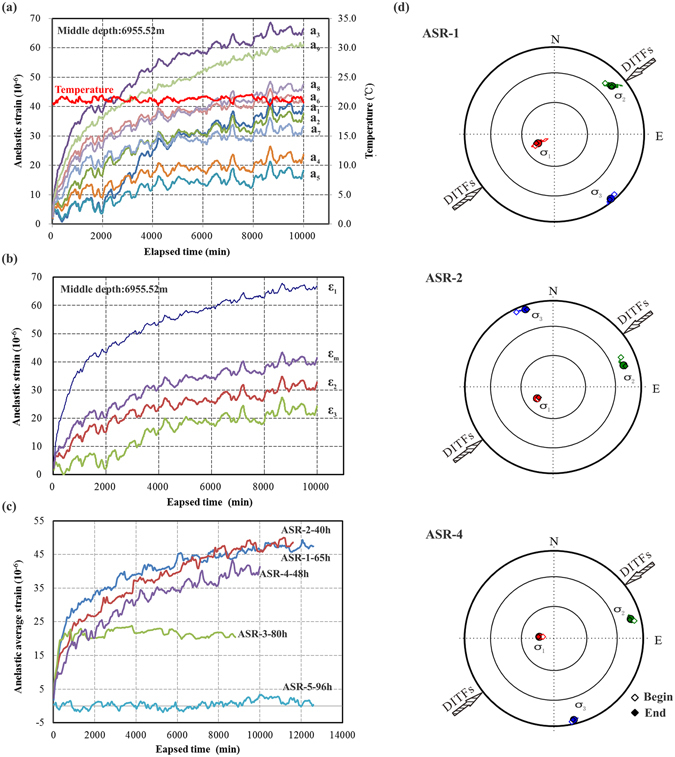
(**a**) Anelastic recovery strains and temperature curves of ASR-4 (6955.52 m mid-depth) in nine directions (“a_1_–a_9_” indicate the directions of longitudinal anelastic strain illustrated in Fig. 4(a), (**b**) The three principal and mean strains versus time curve of ASR-4, (**c**) The mean strains of ASR-1 to ASR-5 versus time. Labels ‘ASR-2-40h’ indicates the strain curve of ASR-2 sample measured starting at 40 hours from stress release, (**d**) Equal-area, lower hemisphere projections of the three principal stresses orientations of ASR-1, ASR-2 and ASR-4. The arrows indicate the azimuths of DITFs observed in the SN-X well.

As mentioned above, a good-quality anelastic recovery strain curve is the key to obtain reliable *in-situ* stress information as well as the validity of the assumptions that the rock is homogeneous, isotropic and linear viscoelastic. The anelastic recovery strain results at ~7 km depth are shown in Fig. 2. Figure 2a shows an example of all raw measurements of the anelastic recovery strains for sample ASR-4 (6955.41–6955.64 m) along with the temperature recorded during the strain measurement. The temperature curve shows about a ± 1 °C fluctuation caused by the relatively large day/night temperature variation in the Tarim Basin. Accordingly, the recovery strain data is also showing fluctuations that has a negative correlation with the temperature fluctuation. This is consistent with the fact that the temperature coefficient of the strain gauges is −11 × 10^−6^/°C and the linear thermal expansion coefficient of ASR samples is about 7 × 10^−6^/°C^16, 17^, which would lead to a negative correlation between strain and temperature. Thus, results clearly show that the temperature effect did not mask the recovery strain data and the overall trend reflects true mechanical rock deformation in response to the stress release. Figure 2b shows the principal and mean anelastic strain components deduced from the independent strain measurements based on least squares regression. According to the principle of ASR method, ASR measurements should be started as soon as possible after the cores were recovered. However, the time spans are 40–96 hours for the SN-X ultra-deep hole in this study (Table 1). As shown in Fig. 2c, the measurement results of mean strains show that three sets of data are valid but not those from samples ASR-3 and ASR-5. Data from ASR-3 and ASR-5 were invalid probably because the time span of 80 and 96 hours from stress release to the start of measurement was too long to record large enough magnitudes of anelastic recovery strain.

### Magnitude of the principal stresses

Using the same method as previous studies, we estimated the stress magnitudes from the ASR data as follows. We made the same two assumptions made by the relevant literatures that the vertical stress is equal to the density-related gravitational overburden stress^18–20^, and that the ratio of the shear recovery compliance, Jas(t), and volumetric recovery compliance, Jav(t), is constant such that Jas(t)/Jav(t) = 1.56^21^. Under these assumptions, the magnitudes of the three principal stresses (the maximum, intermediate and minimum principal stresses σ_1_, σ_2_ and σ_3_, respectively) could be determined from the measured anelastic recovery strain values of the three core samples. Because the original pore pressure in the ASR core samples are unknown, the principal stress magnitudes were calculated as a range of possible values based on the upper and lower limit of the pore pressures. The upper bounds of the pore pressures were suggested to be 88.7, 90.4 and 125.2 MPa (corresponding to mud density 1.41, 1.42 and 1.80 g/cm^3^, respectively) for samples ASR1, ASR2 and ASR4, respectively, from the mud weight adapted during the drilling process to stabilize the well by the managed-drilling pressure technology^22^ (Table 2). The lower bound of the pore pressure was considered to be the hydrostatic pressures which are 62.9, 63.7 and 69.6 MPa for the three depths of the ASR samples, respectively (Table 2). The results show that the uncertainty in the pore pressure did not have drastic influences on the principal stress magnitudes estimated from the ASR method, and stress states predicted from both the upper and lower bound of the pore pressure stayed within the normal faulting stress regime for all 3 samples. The relationship between the three principal stresses and the vertical stress is consistent, being σ_1_ > σ_v_ > σ_2_ > σ_3_ for all the three samples suggesting a consistent stress regime in the studied depth interval (Table 2).

**Table 2:** Orientations and magnitudes of the *in-situ* stresses determined by ASR measurements.

Sample No.	Three-dimensional principal stresses														σ_*v*_ (MPa)
	Orientation (°)						Assuming lower bound of pore pressure (MPa)				Assuming upper bound of pore pressure (MPa)				
	σ_1_		σ_2_		σ_3_		Pp.	σ_1_	σ_2_	σ_3_	Pp.	σ_1_	σ_2_	σ_3_	
	Tr.	Pl.	Tr.	Pl.	Tr.	Pl.									
ASR-1	241	73	49	16	140	4	62.9	165.3	146.2	127.9	88.7	164.9	150.4	136.5	163.6
ASR-2	234	72	72	17	341	5	63.7	168.7	139.6	103.4	90.4	167.7	146.9	121.7	165.6
ASR-4	278	77	75	12	166	5	69.6	183.0	143.2	132.0	125.2	182.3	159.1	153.7	180.8

Tr. demotes trend, Pl. demotes plunge and Pp. demotes pore pressure.

**Figure 3:**
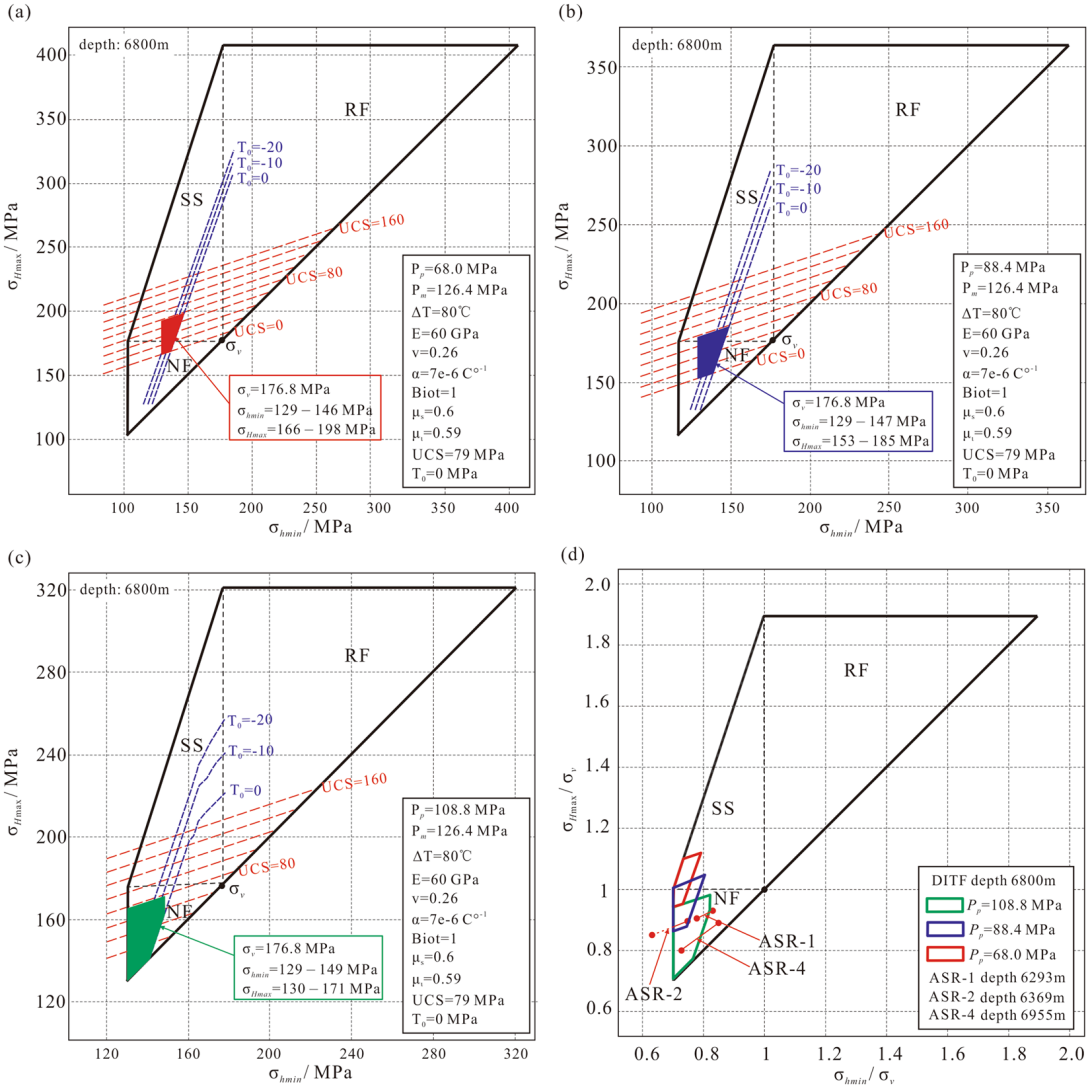
Stress polygons used to constrain the magnitudes of *in situ* horizontal stresses when DITFs are present and breakouts are absent at 6800 m depth with (**a**) the lower bound pore pressure of 68.0 MPa, (**b**) the middle pore pressure of 88.4 MPa, (**c**) the upper bound pore pressure of 108.8 MPa and (**d**) The σ_v_-normalized results of the DITF and ASR methods. The green, blue and red polygons indicate the range of normalized stress magnitudes constrained by the DITFs assuming upper, middle and lower bound pore pressure values, respectively. Red circles are the normalized results from the ASR method assuming lower and upper bound pore pressure values. NF, SS and RF denote normal, strike slip and reverse faulting stress regimes, respectively. The variables *P*_*p*_, *P*_*m*_, *T*, and etc. in the insets are explained in the Method section.

Figure 3a–c show the stress polygons used to constrain the magnitudes of the horizontal *in-situ* stresses at 6800 m depth using pore pressure values 68.0, 88.4 and 108.8 MPa, corresponding to the lower bound (hydrostatic pressure), middle (the average of lower and upper bounds), and upper bound (the mud pressure) values, respectively. Presence of the DITFs requires the stress state to be on the left side of the blue contours representing equilibrium between the minimum borehole circumferential stress, σ_θθ_, and rock tensile strengths, T_0_. Several contours are drawn for tensile strengths corresponding to 0, −10 and −20 MPa. However, we see that the influence of T_0_ variation is not significant, therefore hereon carry out the discussion assuming *T*_*0*_ = 0 MPa. The absence of breakouts also requires the stress state to be below the red contours representing equilibrium between the maximum σ_θθ_ and the rock *UCS* (unconfined compressive strength) = 79 MPa^23^. The magnitudes of σ_*hmin*_ is further constrained to be between 129–149 MPa from the instantaneous shut-in pressures (ISIPs) observed in hydraulic fracturing experiments conducted in a nearby well in the same area of the Tarim Basin^24^, and the vertical stress σ_*v*_ is 176.8 MPa calculated from the density log. The stress states constrained based on the different pore pressure scenarios varied from a normal faulting stress regime in the upper limit pore pressure case (Fig. 3c) to a stress state around the boundary of normal and strike slip faulting stress regimes in the lower limit pore pressure case (Fig. 3a). Overall, the stress regimes fall within the normal or near normal faulting stress regime. The presence of an over-pressured gas reservoir at 7000 m depth gives us plausible reasons to believe that the pore pressure is not at the lower limit case but rather greater than that, thus the *in-situ* stress state is likely in the normal faulting regime.

Figure 3d shows the results of constrained σ_*Hmax*_ and σ_*hmin*_ magnitudes normalized by σ_*v*_ at different depths. The green, blue and red polygons indicate the range of normalized stress magnitudes for the upper, middle and lower bound pore pressure magnitudes, respectively. The σ_v_-normalized results from the ASR method are also shown by the red filled circles which all lie in the normal faulting stress regime. The stress states suggested from both methods are in good agreement.

### Orientation of the principal stresses

**Figure 4:**
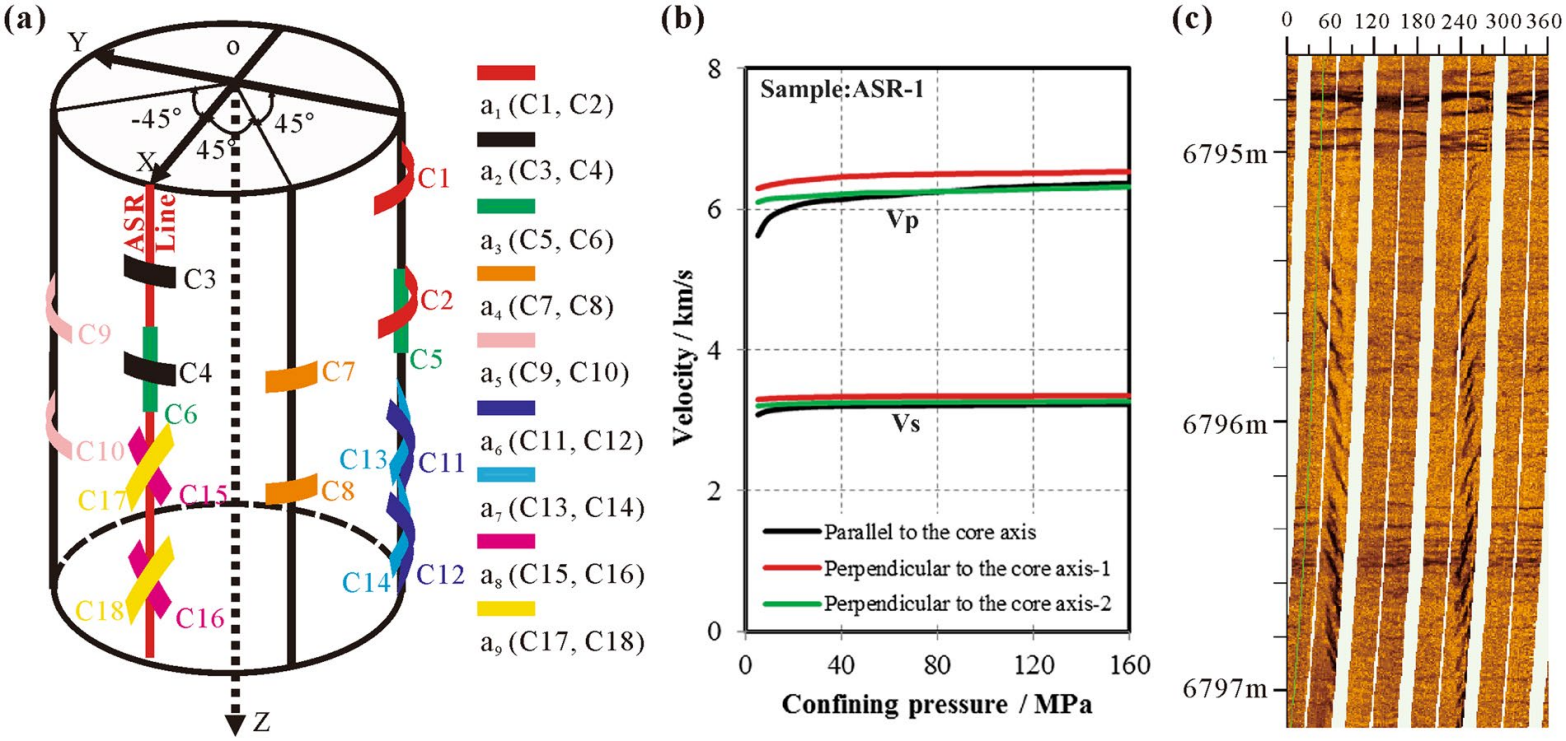
(**a**) Layout of the strain gauges used for onsite ASR measurement, (**b**) Measured compressional and shear wave velocities versus isotropic confining pressure. Specimens were cored from the whole core ASR sample (ASR-1) in three orthogonal directions with one having its axis parallels to the core axis, (**c**) Electrical resistivity images of the borehole wall with en-echelon drilling-induced tensile fractures (DITFs) cutting across horizontal bedding planes in the rock. The tensile fractures occur 180° apart from each other, in the direction of the maximum horizontal stress. Horizontal axis is the clockwise azimuth from North.

The orientations of the reference line on the cores (ASR Line of Fig. 4a) were successfully reoriented to the geographic reference frame by viscous remnant magnetization analysis^25^ using five to nine specimens from each ASR cores. Figure 2d shows the three-dimensional principal stress orientations determined based on the results of ASR measurements carried out on the cores from different depths. The results show that the orientations of the maximum principal stress, σ_1_, are sub-vertical, plunging between 72°–80°, and the intermediate and minimum principal stresses are sub-horizontal (Table 2). DITFs were also observed in the borehole image log (Fig. 4b) at 6400–6900 m depth, within the depth range of ASR cores. The azimuths of the maximum horizontal stress inferred from these DITFs are about 67° East from North^26–28^ (Fig. 2d), which falls within the range indicated by the ASR method.

## Discussion

### The state of stress at ~7 km depth in the central Tarim Basin and its implication

This paper presents results from a rare ultra-deep stress measurements at ~7 km depth which is certainly the deepest measurement made within the Tarim Basin and the entire Qinghai-Tibet Plateau area. The results from both the ASR and DITF methods consistently show that the maximum principal stresses (σ_1_) are close to vertical and the intermediate and minimum principal stresses (σ_2_) and (σ_3_) are approximately horizontal. The state of stress is thus in the normal faulting stress regime at ~7 km depth in the SN-X well.

The present-day state of stress around the Tarim Basin is dominated by a regional compressional tectonic stress field by the Himalayan collisional orogeny^2, 29^. Therefore, it may seem that our results are in conflict with the regional tectonic background. However, extensional structures also formed within the Tarim Basin from Proterozoic to Cenozoic^4, 5^, which have been suggested to be linked to a series of geological events such as the breakup of the Rodinia supercontinent, stress relaxation after the Kunlun Caledonian collisional orogeny, and the tectonic escape with a certain degree of rotation triggered by the far-field effect of the Himalayan collisional orogeny^4, 5, 29^.

The large scale (~600 km) cross-section in Fig. 1c clearly shows that thrust faults close to the mountain ranges are cutting through young sediments (even Quaternary) suggesting recent thrust faulting environment. But at the center of the basin, the thrust faults only reach up to the Permian sediments. Moreover, a clear normal fault sequence is developed at the center of the basin which mainly cuts through the Triassic formations and the bottom of the Jurassic formation, but not through the middle- and late-Jurassic and the Cretaceous sediments (Fig. 1b). These observations suggest that the central region of the basin was under normal faulting stress environment in early Jurassic and Triassic periods. Therefore, somehow between Permian and Jurassic period, the stress state at the center of the basin transitioned from a thrust to a normal faulting environment, whereas it remained thrust faulting at the perimeter of the basin. In this study, we provide direct evidence that the present-day stress state in the depression of the lower Ordovician stratum is in the normal faulting stress regime. This suggests that the stress environment in the central Tarim Basin may have remained extensional through the period from the middle Jurassic to today without any intensive tectonic activities and re-construction of the stress regime. The transparent seismic section, absent of any faulting structures, in the shallower Cretaceous and Paleogene sediments (Fig. 1b) also supports this interpretation. The *in-situ* stress state is one of the fundamental boundary conditions in any geomechanical and geodynamics analyses. The *in-situ* stress results revealed in this paper will be a basis for future studies of the extensional structures found in the Tarim Basin.

### Applicability of the ASR method to ultra-deep hard rocks

Lin *et al*.^16^ has shown that the ASR method can be applied in hard rocks within the range of about 2400–4500 m depth. This study is the first successful application of the ASR method to ultra-deep hard rocks at 6293–6955 m depth, where anelastic strain measurements only started after 40–65 hours from the time the cores were released from the *in-situ* stress. The anelastic strain curves from the onsite measurements were of good except for the ASR-3 and ASR-5 samples. The good agreement of the results from the ASR and DITF methods shows that the ASR method is also applicable and effective for stress measurements at ultra-depth. The fact that *in-situ* temperature and stress/pressure magnitude does not impose any technical depth limit to the ASR method especially makes it a strong option for stress measurements at ultra-depth. From the micritic limestone and dolomitic limestone samples used in this study, effective information of anelastic recovery strain were recovered when strain measurements started less than 80 hours after the cores were released from the *in-situ* stress. However, for ASR-3 and ASR-5 samples whose strain measurements started after 80 and 96 hours from the stress release, this was not the case. The strain recovery in the ASR-3 stopped after an initial sharp increase, and the ASR-5 sample exhibited almost no strain recovery (Fig. 2c). This time threshold for ASR measurements should certainly vary according to various factors such as rock types and stress magnitudes. For instance, soft rocks typically have bigger anelastic recovery strain than hard rocks when subject to the same stress release^16, 30^, but whether the time threshold of the anelastic recovery deformation for soft rocks are longer or shorter than hard rocks should also depend on the viscoelastic time constant that governs the time scale of the strain recovery, which is not explored in detail yet.

### Comparison between the ASR and DITF methods

Although the ASR and DITF methods generally yielded *in-situ* stresses that agree with each other, there were certain discrepancies between the two stress results. With regards to the orientation of the principal stresses, we observed symmetrical en-echelon inclined DITFs with consistent inclination throughout the observed depth interval, which suggests that the maximum principal stress (σ_1_) is slightly but consistently deviated to the southeast direction from vertical. However, ASR results show that the azimuth of the maximum principal stress (σ_1_) varies with depth and not necessarily consistent with the direction inferred from the inclined DITFs. Since DITFs form under the direct influence of the current *in-situ* stress, we believe that the deviation direction from the DITF is more reliable. The deviation direction was actually in the southeast direction but the ASR method was not able to capture this direction. With regards to the magnitude of the principal stresses, the ASR method estimates the full three-dimensional stress tensor and its magnitude based on the overburden stress and the ASR compliances of the core materials. But for the DITF method, the workflow require many additional parameter inputs compared to the ASR method, and furthermore, only provides a range of maximum horizontal stress magnitude (Fig. 3a). Given the limitations of each method, a combination of the ASR and DITF (or breakout) methods should be used, when available, for the most reliable estimate of *in-situ* stresses.

## Methods

### ASR method for estimating *in-situ* stress state

The ASR method was proposed by Voight^31^ based on the empirical observation that the anelastic strain recovered in a rock is proportional to the pre-existing stress state when the rock is homogenous and rheologically isotropic. Initially, it was proposed as a two-dimensional *in-situ* stress measurement method, usually combined with the assumption that the vertical stress is also one of the three principal stresses. Subsequently, Matsuki^14^ extended the technique to a three-dimensional method and showed that it could be used to estimate the full stress tensor with the assumption that the rock is homogenous, isotropic and linearly viscoelastic, and that the shear and volumetric compliances are independent. The orientations of the three principal *in-situ* stresses are consistent with the orientations of the three principal anelastic recovery strains for isotropic viscoelastic materials. Thus, the orientations of the principal stresses can be determined from the anelastic recovery strain data measured in at least six independent directions. Also, the magnitudes of the stress components can be recovered as a function of the anelastic recovery strains (mean and deviatoric strain components), material constants (shear and volumetric compliances, linear thermal expansion coefficient) and pore pressure^16, 32^. In this paper, we conducted ASR measurement based on the principle suggested by Matsuki^14^ and employed the same apparatus and procedures used by Lin *et al*.^16, 30^. As shown in Fig. 4a, the Z-axis was parallel to the borehole axis. Six cross-type wire strain gauges and six single-type gauges were mounted on the ASR sample surface to measure the anelastic recovery strain in nine directions, including six independent directions.

In order to verify the homogeneity and isotropy of the ASR samples, elastic velocities of the ASR samples were measured in a triaxial apparatus at *in-situ* stress magnitude conditions (Autolab-2000c, New England Research Inc.). Cylindrical specimens for these velocity tests were cored in three orthogonal directions with the axis of the specimens being either parallel to or perpendicular to the bedding of the larger ASR sample. The measurements were made at room temperature on cylindrical specimens of 2 inch length and 1 inch diameter. Figure 4b shows the measured compressional and shear wave velocities as a function of isotropic confining pressure. The results demonstrate that the elastic velocities in three orthogonal directions are fairly close to each other with differences less than 3% when the confining pressure was higher than 30 MPa. The results verify that the cores used for ASR *in-situ* stress measurements are nearly homogenous and isotropic. Slightly greater difference at low pressure is present though that could be caused by the presence of microcracks in some samples.

### DITF method to constrain ***in-situ*** stress state

It is well known that stress concentrations are produced around the borehole after drilling which can be described by the Kirsch equations^33^ for cylindrical boreholes. Wellbore failures will occur if the perturbed stress field around the wellbore meets the failure criterion of the rock^26, 34^. Figure 4c shows high resolution electrical resistivity images of the borehole wall clearly indicating the presence of drilling-induced tensile fractures (DITFs). All DITFs were also found to be inclined with respect to the borehole axis forming an en-echelon pattern. These en-echelon tensile fractures are clearly drilling-induced (Fig. 4c) because natural fractures were not present in the cores and the tensile fractures clearly cut across the pre-existing planes of weakness^35^.

**Figure 5:**
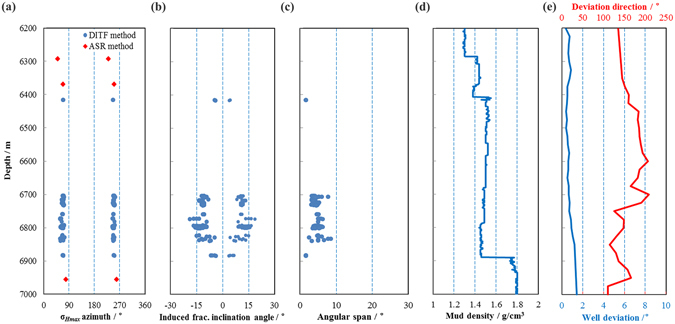
(**a**) Azimuth of σ_*Hmax*_ by the DITF (blue circle) and ASR (red diamond) with respect to depth, (**b**) Distribution of DITF inclination angle with respect to depth, (**c**) Distribution of DITF angular span with respect to depth, (**d**) Drilling mud density at surface with respect to depth, (**e**) Well deviation and deviation direction with respect to depth.

The statistics of the inclined DITFs indicate that the DITFs are symmetrically distributed around the borehole wall, and that the azimuths of the maximum horizontal stress inferred from DITFs are about 67° East from North. Thus the maximum horizontal stress azimuths are roughly consistent between the DITF and ASR methods (Fig. 5a). Nearly all the DITFs in the interval are inclined in a preferred direction with the inclination angles, ω, ranging between 4–18° (Fig. 5b). The azimuthal span of the inclined DITFs ranges between 2–8° (Fig. 5c). Figure 5d shows the inlet mud density parameters from the drilling engineering data. As shown in Fig. 5e, the deviation of the borehole axis from vertical was smaller than two degrees, which is normally regarded to have indistinguishable effect on the inclination of DITFs^28, 35^. Therefore the inclination of DITF is due to the deviation of the principal stress axis from the vertical direction, not the borehole itself.

### Magnitudes of principal stresses by the DITF method

Given the Kirsch equation below, we can estimate the magnitude of the far-field principal stresses if all other parameters are constrained.

1 $${\sigma }_{\theta \theta }={\sigma }_{Hmax}+{\sigma }_{hmin}-2({\sigma }_{Hmax}-{\sigma }_{hmin})\cos 2\theta -({P}_{m}-{P}_{p})-{\alpha }_{T}{\rm{\Delta }}T\frac{E}{(1-v)}$$

In Equation (1), σ_*Hmax*_ and σ_*hmax*_ are the magnitudes of the (total) far-field maximum and minimum horizontal stresses. *θ* is the circumferential position relative to the azimuth of σ_*Hmax*_, σ_*θθ*_ is the effective circumferential stress, *P*_*m*_ is the downhole mud pressure exerted on the wellbore wall by the drilling mud, *P*_*p*_ is the pore pressure, α_*T*_ is the linear thermal expansion coefficient, ΔT is the difference between mud and formation temperatures, E and ν are Young’s modulus and Poisson’s ratio, respectively. It is important to note that Equation (1) applies only when the borehole axis is aligned with one of the principal stresses. As was seen above, this is not the case in our study where the principal stress axis is slightly deviated from the vertical direction. However, if the misalignment between the borehole axis and a principal stress is small, one can still apply the Kirsch equations to constrain the magnitude of stress. For instance, Sone^28^ showed that the misalignment of the borehole axis and the principal stress up to 15 degrees had little effect on the stress magnitudes deduced from the wellbore failures. Thus, we assume that the same is true for this study and we apply Equation (1) to constrain the magnitude of the maximum horizontal stress. If σ_*θθ*_ from Equation (1) becomes negative, the tensile stress pulls apart the borehole wall to form a vertical DITF, assuming the lower bound tensile strength of 0 MPa^35^. As a representative example, we constrain stress state at 6800 m depth because it locates between the ASR samples and abundant high quality DITFs were observed at that depth (see Fig. 5a).

The basic parameters required to constrain the maximum horizontal stress magnitude at 6800 m depth are listed in Table 3. The vertical stress (σ_*v*_) was calculated from the density log. The minimum horizontal stress was estimated from the instantaneous shut-in pressures (ISIP)^34^ observed during a hydraulic fracturing conducted in a nearby well. The downhole mud pressure was taken as the sum of the maximum mud pressure and 95% of the excess pumping pressure at the surface used to circulate the mud^11^. The mud outlet temperature and the formation temperature were obtained by onsite logging. The coefficient of linear thermal expansion of limestone was 7 × 10^−6^/°C, which was measured by strain gauges using equipment similar to the equipment used for ASR measurements^36^. The Young’s modulus *E* and Poisson’s ratio *v* are derived from P and S wave elastic velocities measured in three orthogonal directions. The *UCS* came from past measurements from the same formation^23^. As pressure difference between *P*_m_ and *P*_*p*_ exist, the contribution of radial stress σ_*rr*_ (σ_*rr*_ = *P*_*m*_ − *P*_*p*_) to the rock compressional strength should not be ignored^35^, and the internal friction coefficient of limestone was taken as 0.59^37^.

**Table 3:** Parameters used to constrain σ_*Hmax*_ magnitude and their sources.

Parameter	σ_*v*_ (MPa)	σ_*hmin*_ (MPa)	P_*p*_ (MPa)	P_*m*_ (MPa)	Biot’s Coefficient	Mud outlet Temp. (°C)	Formation Temp. (°C)	α_*T*_ (e^–6^/°C)	E (GPa)	ν	UCS (MPa)	Internal friction	Sliding friction
Depth 6800 m	176.8	129–149	68–108.8	116.2	1	58	178	7	60	0.26	79	0.59	0.6
Source	density log	Li *et al*., 2007^24^	Log data	Mud log data	Common estimate	Log data	Log data	Sun, 2014^36^	Velocity test	Ding, *et al*., 2011^23^	Yin *et al*., 2017^37^	Common estimate	

Unfortunately, the formation pore pressure has not been measured in SN-X well and we can only infer ranges of possible pore pressure values from other information. The drilling engineering data showed that the mud density used for drilling was 1.45 g/cm^3^ when the drilling bit passed 6800 m depth on Sep 5, 2014. The drilling operation in this interval continued down to 6890 m depth by Sep 15, 2014 with a mud density of 1.60 g/cm^3^. Then downhole logging including FMI imaging was conducted on Sep 16, 2014. Thus, the images revealing the DITFs were obtained ~11 days after the drilling reached 6800 m. During this 11 day time period, the pore pressure around the borehole would have increased towards equilibrium with the mud pressure, and may have been anywhere between the hydrostatic pore pressure of 68 MPa and the upper limit of 108.8 MPa constrained by the mud weight. Therefore we constrained the stress magnitudes based on the lower (hydrostatic), middle and upper (mud weight) bounds of the pore pressure (Fig. 3a–c).

In this study, the outlet mud temperature and the undisturbed temperature of the formation at 6800 m depth were about 58 °C and 178 °C, respectively. However, no measurements exist for the downhole temperature of the circulating mud which is necessary to constrain the ΔT in Equation (1), and thus necessary to evaluate the magnitude of the thermal stress. The downhole temperature of the circulating mud is a difficult parameter to constrain, as there are many factors influencing the mud temperature including the geothermal gradient, borehole diameter, circulation time, mud flow rate, specific heat and thermal conductivity of the surrounding rock, cemented casing depth, and etc^38^. Brudy *et al*.^11^ estimated the temperature difference between the undisturbed formation temperature and the circulating mud temperature during drilling to be about 80 °C at 6800 m depth in the KTB main borehole whose geothermal gradient is 27.4 °C/Km. In the SN-X well, the drilling mud at surface temperature was pumped into the borehole and the geothermal gradient was ~27 °C/Km, which is almost the same with the geothermal parameters of the KTB main borehole^11^. Thus we used the same value as Brudy *et al*.^11^, a temperature difference of 80 °C at 6800 m depth, as the maximum amount of cooling the rock experienced. The resulting thermally-induced tensile stress is about 45 MPa. Calculations show that changes in this temperature difference value by, for instance, ±20 °C has a small impact (less than 4 MPa) on the estimated magnitude of σ_*Hmax*_. Thus, error in our estimate of the thermal stress has a minor impact on the estimated magnitude of σ_*Hmax*_, and does not change the conclusions of this study.
